# The Human Brain; Its Configuration, Structure, Development, and Physiology: Illustrated by References to the Nervous System in the Lower Orders of Animals

**Published:** 1837-10

**Authors:** 


### ART. XV.

*The Human Brain; its Configuration, Structure, Development, and Physiology: illustrated by References to the Nervous System in the lower Orders of Animals.* By SAMUEL SOLLY, Lecturer on Anatomy and Physiology in St. Thomas's Hospital, &c. *With twelve Plates.—London*, 1836. 8vo. pp. 492.

CIRCUMSTANCES, which it is unnecessary here to mention, have prevented our noticing this book sooner; and we are even now under the necessity of contenting ourselves with a briefer account of it than its merits may justly lay claim to. By the production of this work, Mr. Solly has entitled himself to the gratitude of a numerous class of readers, the students in anatomy, for whose use and advantage his book is specially intended. Fully impressed with the insufficiency of that method of dissecting the brain which consists in removing its substance by successive layers, proceeding from above downwards, and in pointing out the different forms which are thus disclosed, Mr. Solly advises to commence with the spinal cord, and to trace its constituent parts through the various modifications of form and of structure which they undergo, until they are expanded into the hemispheres of the brain and the lobes of the cerebellum. He rejects the absurd or inapplicable names given to many parts of the brain by anatomists, to whom the uses of these parts were unknown, or who attached undue importance to accidental forms; and he substitutes for them terms derived either from the well-understood functions or from the shapes or other physical relations of the parts requiring distinct appellations.

The work consists of eight parts: the first part treats of the comparative anatomy of the Nervous System, commencing with the Ascaris, and passing on through the Articulata, Mollusca, Fishes, Birds, up to the Mammalia. The different forms of the nervous system throughout this series of animals are pointed out; and the gradual approach to that state of concentration of its central parts, by which the higher classes, and most remarkably the human subject, are distinguished, is placed in a prominent and judicious point of view. Various opinions as to the uses of the cineritious and medullary portions of the nervous mass are compared with each other, and the author's view of this subject is stated to be "that the peculiar power of the nervous system resides in the cineritious portion, and that the office of the medullary is simply that of a conductor." The literary history of the Sympathetic Nerve, from Galen to Braschet, is briefly detailed; and the author proposes to apply to this part of the nervous system in man the designation of "*Cyclo-ganglionic* system, as corresponding in its mere anatomical arrangement with the nervous system of the cyclo-gangliated or molluscous division of the animal kingdom."

The second part of the work describes the form of the Vertebral Canal, the Membranes of the Brain, and the Sinuses; it also contains some excellent directions for the removal of the brain and spinal cord from the body; it treats of the configuration of the different parts of the former organ, and briefly of its internal structure. The author adheres to the ordinary division of its substance into medullary, or white, or fibrous, and cineritious or pulpy; and admits the existence of a fine cellular membrane pervading all its parts, as demonstrated by Dr. Macartney. In his description of the forms of the spinal cord, we were much surprised to find him stating that it gradually tapers "off to a point opposite the second lumbar vertebra, where it terminates in a *single nerve*." This termination, although regarded by old anatomists as a nerve, and called by some of them "the fortieth pair," is now well known to be a ligament, extremely elastic, and conjoined with two branches of vessels by which it is accompanied to its attachment to the os coccygis: it is analogous to the ligamentum denticulatum.

The third part contains the "Dissection of the Human Brain and Spinal Cord," and is extremely well worth the attentive perusal of the student. In the plates belonging to this part of the work are given some instructive views of sections of the cord and of the medulla oblongata, showing the relation which exists between the cineritious and the medullary portions in each of these organs. Mr. Solly regards the olivary bodies and the posterior pyramidal bodies as the ganglia, the former of the function of respiration, the latter of the sense of hearing.

"The view I am inclined to take of the character of the parts comprising the medulla oblongata is simply this: in addition to the columns for motion and sensation, there are here deposited, and imbedded to a certain extent in its substance, four ganglia, two on each side. The most anterior of these are the ovoid bodies, which derive the name of *olivary* from their form. We have already observed their analogues in moths giving origin to the par vagum, in fish, to the branchio-gastric nerves; in like manner, in man they seem to me to be the appropriate ganglia of the pneumo-gastric nerves. The posterior ganglia are found in the fissure at the back part of the cord, which is known by the absurd name of fourth ventricle. They form two projections of a pyramidal figure, and are usually designated the *posterior pyramidal bodies.* In these bodies terminate the auditory, or eighth pair of nerves. These we have also remarked in the fish, under the title of tubercles of the fourth ventricle." (P. 145.) . . . . "In support of the opinion that the respiratory muscles are dependent on the corpus olivare for their stimulus to contraction, the results of two or three experiments may be related. A section of the spinal cord made above the origin of the intercostal nerves simply annihilates, as regards the respiratory movements, the power of the intercostal muscles. A section above the phrenic nerve induces paralysis of the diaphragm; while a section exactly at the origin of the par vagum, and therefore through the corpus olivare, occasions a total cessation of every respiratory movement, and instant death. If the section, however, be made above the corpus olivare, then the whole of the respiratory movements take place as usual. Is it not, then, from this point, and this only, that they draw their power of motion? A section of the par vagum produces no such effect: the section must destroy the corpus olivare before total interruption to the respiratory action can take place." (P. 147.)

The following are his remarks on the anterior columns, or motory tracts of the cord.

"Of the fibres which run from the antero-lateral columns to the cerebellum, there are evidently two sets; one superficial, and one deep. The *superficial* may again be divided into two sets: the first cross the surface of the cord immediately below the corpus olivare, and may generally be seen without dissection: they are more distinct in the sheep, bullock, and horse than in man, in whom they form a very thin layer emanating from the corpora pyramidalia; and I have no doubt that they actually decussate with their fellows of the opposite side, forming, in fact, part of the apparatus of decussation, though I have not yet positively ascertained the fact. The *second* of the superficial set of fibres takes the same direction; only, instead of crossing the cord immediately below the corpus olivare, they run to the inner side of the corpus olivare, and then, ascending to the cerebellum, they form the outer part of the corpus restiforme. The deep set of fibres from the antero-lateral columns of the cerebellum are the most posterior of the whole mass of fibres composing this portion of the spinal cord. They are separated from the posterior columns by the posterior fissure from which the posterior roots of the spinal nerves emerge: this fissure they cross in their passage to the cerebellum, obliterating it entirely. Thus it will be perceived that one portion of the antero-lateral columns, . . on reaching to within a small distance of the corpus olivare, splits into three sets of fibres; one, and the most anterior of which, passes through the pons Varolii, as will be presently described, and may be designated the *cerebral* fibres of the anterior columns. A second set, which may be entitled the *superficial cerebellar* fibres of the anterior columns, passing over the surface of the medulla oblongata, are usually seen without dissection. . . The third, or *deep cerebellar* fibres of the anterior columns, proceeding in company with those of the posterior columns, form about a fourth part of the whole diameter of the restiform bodies." (P. 155.) . . . . "The fibres just described as connecting the antero-lateral columns of the cord with the cerebellum are peculiarly interesting, when viewed in relation to the functions of the cerebellum. For . . the experiments of Flourens, Bouillaud, Magendie, and others, and the numerous cases on record in which disease of the cerebellum has been followed by paralysis, all tend to prove that the cerebellum is in some way or other connected with the regulation of muscular action." (P. 162.)

The course of the remaining fibres of the motor tract through the pons Varolii and the corpus striatum, or *anterior cerebral ganglion*, and their termination at the convolutions, or *hemispherical ganglion*, having been described, the author proceeds to notice the commissures. The "fornix" is named by him "*the inferior longitudinal commissure*," whose office is to connect the anterior and posterior parts of the same hemisphere, as the transverse commissures do those of the opposite hemispheres. *The superior longitudinal commissure* is that band of fibres which runs, in each hemisphere, above the *great transverse commissure*, or corpus callosum, on the edge of the longitudinal fissure. Its use is supposed to resemble that ascribed to the *superior longitudinal commissure.* The pineal gland and its peduncles form the *pineal commissure;* while the "processus e cerebello ad testes," with the interposed "valve of Vieussens" have been named the *oblique* or *inter-cerebral commissure.* In describing this structure, Mr. Solly states that it is composed of medullary fibres: whereas, in truth, the middle portion, or "valve of Vieussens," contains a large portion of cineritious substance, and, if examined with a magnifying glass, is found to be a most delicate ganglion. In this part of his work the author introduces the description of the cerebellum given by Reil, in the Archiv. für Physiologie; a description which we must characterize as tiresomely and uselessly minute, fitted to confuse the understanding of a student, and incapable of affording useful knowledge to a more mature enquirer. We are surprised that the taste and judgment which lead Mr. Solly to reprehend and reject a system, "requiring him (the student) to burthen his memory with fanciful and unmeaning names," . . . "and puzzling his brain with the absurd titles of hippocampus major and minor, pes hippocampi, tænia hippocampi, cornu Ammonis, &c. &c.," have allowed him to present to his readers a description which assigns to the parts of the cerebellum the equally absurd titles of *valley, pyramid, nodule, spigot, flocks,* and *wings.* We hope that, in the next edition of his useful work, he will, undeterred by the doubtful authority of a name, substitute for this unprofitable detail of minute and uninteresting localities, a clear and simple account of the cerebellum, written by himself; tracing this organ from its earliest appearance in the animal kingdom, and describing with accuracy its internal, that is—its essential structure.

Lastly, the passage of the column or tract of sensation is traced in part to the cerebellum, and in remainder through the pons Varolii, and the thalamus, or posterior cerebral ganglia, to the hemispherical ganglion of the brain.

In the fourth part is to be found an enumeration of the Cerebral Nerves, with a description of their connexions to the central parts of the nervous system. The fifth part is devoted to the Cerebral Circulation. Neither of these requires any observations from us. The sixth part contains a history of the Development of the Brain, taken from the writings of Tiedemann, Serres, the Wenzels, &c. The seventh is on the Physiology of this organ, and consists of the "Report made to the Royal Academy of Sciences of the Institute," on M. Flourens' well-known memoir on the Properties of the Nervous System; of some further experiments of M. Flourens; and of extracts from publications on the same subject, by Messrs. Bouillaud, Magendie, and Foville.

The eighth and last part is designated "Physiological Inferences from Pathological States," and contains the details of several cases of injury or disease of the brain, derived partly from the observation of the author, but principally from other sources. In this part of his work Mr. Solly endeavours to illustrate the functions of particular parts of the brain by connecting the symptoms observed during life with the organic changes discovered after death. The opinions of various eminent pathologists are noticed and compared; and the conclusions of the author do not materially differ from those generally held on this subject.

From the brief review which we have given of Mr. Solly's book, our readers must have perceived that it contains much valuable matter, presented generally in an agreeable and intelligible form, and leading the student gradually onwards from a notice of the nervous system in the simplest class of animals, to the consideration of its complicated functions and pathological states in the human subject. The views contained in the third part, on the Dissection of the Brain, are remarkable for their simplicity and good sense, and deserve the especial attention of the anatomical student.

Mr. Solly's work does not put forward strong claims to originality of matter, considerable portions of it consisting of avowed quotations from the writings of others, and of statements of the opinions and discoveries of other authors in a condensed or modified form. This circumstance, however, does not in the least diminish the utility of the work: every writer, who successfully devotes his time and talents to the careful arrangement of the leading facts in any department of useful science, is entitled to much praise. Of this meed Mr. Solly is richly deserving. He has proved himself a learned and a skilful anatomist; and it is no less a point of justice to the anatomical student than to Mr. Solly, to recommend his work in the strongest terms.

